# Encapsulation of Cinnamon Essential Oil for Active Food Packaging Film with Synergistic Antimicrobial Activity

**DOI:** 10.3390/nano8080598

**Published:** 2018-08-06

**Authors:** Ben Niu, Zhipeng Yan, Ping Shao, Ji Kang, Hangjun Chen

**Affiliations:** 1 Department of Food Science and Technology, Zhejiang University of Technology, Hangzhou 310014, China; newben1989@163.com (B.N.); zjgydxnb1989@163.com (Z.Y.); 2 Guelph Food Research Centre, Agriculture and Agri-Food Canada, Guelph, ON N1G 5C9, Canada; jkang12@uoguelph.ca; 3 Institute of Food Science, Zhejiang Academy of Agricultural Sciences, Hangzhou 310021, China

**Keywords:** antimicrobial, nano-porous materials, cinnamon essential oil, bayberry fruit

## Abstract

Porous adsorption, a less powerful adsorptive force than chemical bonds, is based on the physical adsorption of small molecules onto a solid surface that is capable of adsorbing gas or liquid molecules. Antimicrobial permutite composite (containing Ag^+^, Zn^2+^ and Ag^+^/Zn^2+^), starting from Linde Type A-permutite (LTA), was obtained in this research. The permutite samples were characterized by field emission scanning electron microscopy (FESEM), X-ray diffraction (XRD), colorimeter and nitrogen adsorption technique. Cinnamon essential oil (CEO) was encapsulated into Ag^+^/Zn^2+^-permutite. The FT-IR and differential scanning calorimetry (DSC) confirmed that no chemical bond existed between CEO and Ag^+^/Zn^2+^-permutite. The loading capacity of Ag^+^/Zn^2+^-permutite/CEO was 313.07 µL/g, and it had a sustained release effect. The Ag^+^/Zn^2+^-permutite/CEO showed stronger efficacy against *Aspergillus niger* and *Penicillium* sp. than Ag^+^/Zn^2+^-permutite. Ethyl cellulose pads modified by composite antimicrobial particles were applied in the preservation of Chinese bayberry. Compared to the control group, treatment with the Ag^+^/Zn^2+^-permutite/CEO antimicrobial pads resulted in a significantly lower decay incidence. In addition, the amount of migrated silver, zinc and aluminum from LTA was below the legal limit. These results confirmed that the ethyl cellulose pads modified by the Ag^+^/Zn^2+^-permutite/CEO provided an active packaging to control decay of fresh Chinese bayberry.

## 1. Introduction

In recent years, the development of science and technology has brought new functions to packaging [1]. Antimicrobial packaging, one of the active packaging concepts, can be considered to be an extremely challenging technology that could extend shelf life through a mild preservation technique while sustaining the nutritional and sensory qualities of fresh fruits [2]. Nano antimicrobial packaging had a significant impact on sensory, physicochemical, and physiological quality of fresh fruit compared to traditional packaging materials [3,4].

**Figure 1:**
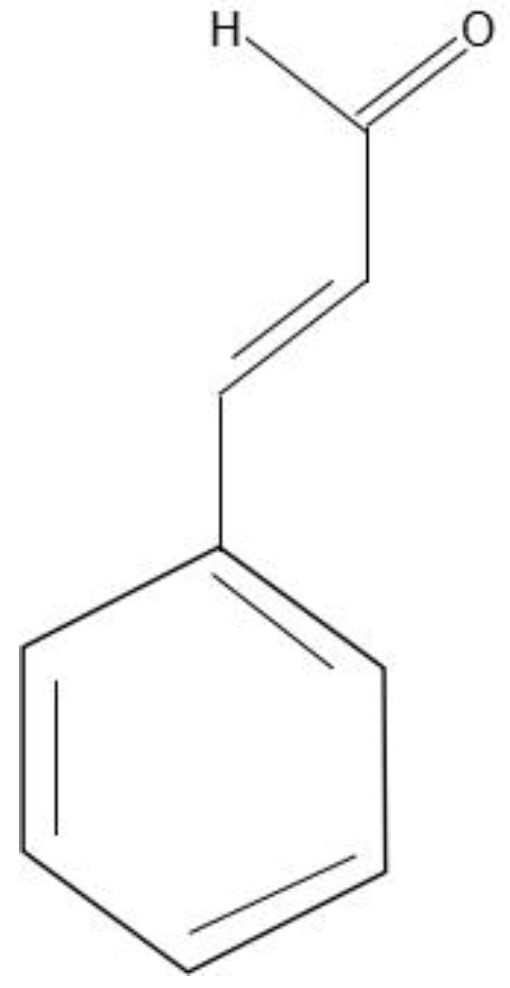
Chemical structure of cinnamaldehyde.

Silver ions have strong antimicrobial capacity against a broad range of microorganisms, such as *Pseudomonas aeruginosa*, *Escherichia coli*, *Scrub typhus* and *Vibrio cholerae* [5]. It has a long-term germicidal effect, low volatility, and low toxicity on eukaryotic cells [6]. Zinc ions are believed to reinforce the antimicrobial activity of silver by interfering with proton transfer and inhibiting nutrient uptake [7]. Permutites are artificial nano-porous alumina silicates composed of silicon, aluminum and oxygen in a framework with cations and water within pores [8]. Silver and zinc ions have been trapped in permutate-type microporous inorganic ceramics, exchanged for cations of permutate, thus expanding the applications of silver in diverse fields [9]. Although silver/zinc zeolite is being extensively used in food and other applications as a key component to control microbial proliferation [10], few studies have reported the practical application for the quality maintenance of fruits and vegetables [11]. Silver/zinc zeolite lacking an effect under high nutrient conditions and humidity was confirmed with other food-associated bacteria [10]. Many experiments have also confirmed that silver/zinc permutite has good stability, but that the antibacterial effect is limited by its diffusion capacity. In addition, silver migration from the packaging has attracted much attention [12]. Cinnamon essential oil (CEO) is a natural essential oil with powerful antimicrobial activity against foodborne pathogens and fungi. The chemical structure of cinnamaldehyde is shown in Figure 1 [13]. However, CEO undergoes heat-induced decomposition at fairly low temperatures (<60 °C), producing benzaldehyde [14,15]. Therefore, the application of CEO to improve the antibacterial properties of packaging materials should avoid high temperatures.

The permutite samples were characterized by field emission scanning electron microscopy (FESEM), X-ray diffraction (XRD), colorimeter and nitrogen sorption technique [16]. The anti-discoloration properties and structure of permutite samples were evaluated. CEO was encapsulated into Ag^+^/Zn^2+^-permutite at 4 °C, and the Ag^+^/Zn^2+^-permutite/CEO release behavior in the air was also evaluated. In this study, the permutite was proved to be Linde Type-A permutite by XRD, and the bimetallic monometallic permutite was prepared by ion exchange. The ethyl cellulose can be combined with efficient plasticizers derived from renewable resources to form supramolecular systems [17]. Then, the ethyl cellulose pads were modified by composite antimicrobial particles. The objective of this study was to evaluate the antimicrobial properties of the pads. The ethyl cellulose pads modified by composite antimicrobial particles were applied in the preservation of Chinese bayberry, and the decay rate and total soluble solids (TSS) were determined over 96 h storage at 20 ± 0.5 °C. The migration security of metal ions (Ag^+^, Zn^2+^, Al^3+^) was evaluated in the simulant using inductively coupled plasma mass spectrometer (ICP-MS). These results confirm that the ethyl cellulose pads modified by the composite antimicrobial particles provided an effective and safe method to control the decay of Chinese bayberry fruit.

## 2. Materials and Methods

### 2.1. Materials

Permutite ((SiO_2_)_x_ (Al_2_O_3_)_y_, particle size ≤ 10 µm), AgNO_3_ (AR, 99.8%), Zn(NO_3_)_2_∙6H_2_O (AR, 99%) and ethyl cellulose (CP) were purchased from Aladdin Chemistry Co., Ltd. (Shanghai, China). Ethanol (AR), acetic acid (AR), agar (AR) and ether (AR) were purchased from Sinopharm Chemical Reagent Co., Ltd. (Shanghai, China). Glucose (AR) was purchased from Guanghua Technology Co., Ltd. (Guangdong, China). Distilled water purchased from Wahaha Group Co., Ltd. (Hangzhou, China). Fresh potatoes were obtained from a local market. Chinese bayberry fruit was obtained from Jinhua, Zhejiang Province, China, in June 2017, and transported to the laboratory at Zhejiang University of Technology, Hangzhou, within 4 h of harvest.

### 2.2. Preparation of the Ion-Exchanged Permutite and Ag^+^/Zn^2+^-Permutite/CEO

The solutions for the cation exchange runs were prepared with AgNO_3_ and Zn(NO_3_)_2_∙6H_2_O using distilled water as solvent. Permutite was treated with different solutions: (a) Untreated, (b) 0.1 mol∙L^−1^ Ag^+^, (c) 0.1 mol∙L^−1^ Zn^2+^, (d) 0.1 mol L^−1^ Ag^+^ and 0.1 mol∙L^−1^ Zn^2+^. Ion exchange runs were carried out contacting 10.0 g of permutite with 100 mL of the corresponding exchange solution. After a contact time of 6 h at 60 °C, the solid was separated from the liquid by vacuum filter. The solid phase was dried in a high-temperature vacuum oven at 80 °C. In consideration of the light sensitivity of silver, the glassware was covered with foil as a precaution during all experimental procedures. Ag^+^/Zn^2+^-permutite containing CEO was prepared by dissolving 3 mL CEO in 4 g Ag^+^/Zn^2+^-permutite at 4 °C for 2 h. Then, the product was washed with ethanol to remove the excess of essential oil. After that, the Ag^+^/Zn^2+^-permutite/CEO was sealed and stored at 4 °C.

### 2.3. Characterization and Measurement

The morphology and chemical analysis of the materials were observed by field emission scanning electron microscope (SU8010, HITACHI, Osaka, Japan) equipped with an energy dispersive X-ray spectroscopy (EDS). Prior to SEM imaging, the permutite samples were coated with platinum (Pt) using a sputter coater under vacuum. The acceding voltage was 15.0 kV and the working distance was 9.1 mm with a magnification of 5000×. The powder XRD diffraction patterns of the materials were acquired at room temperature using a diffractometer with automatic data acquisition, from 10° to 80° 2θ, at a scan rate of 2°/min (X’pert pro, PANalytical, Almelo, The Netherlands).

### 2.4. Anti-Discoloration Property Assays and Nitrogen Physisorption

The chroma values (*L*_0_*, *a*_0_*, *b*_0_*) of ion-exchanged permutite were measured by colorimeter (ColorQuest XE, Hunter Lab, Reston, VA, USA). The permutite samples were treated in a 254 nm UV irradiation box for of 5 cycles of 4 h, and measured again (*L*_1_*, *a*_1_*, *b*_1_*). In hunter whiteness formula, the whiteness of the fully reflected diffuser was defined as 100, and the whiteness of the sample was compared with the whiteness of the fully reflected diffuser. The whiteness of the sample was evaluated by calculating the color difference [18]. The hunter whiteness formula was calculated using Equation (1):

(1) $$W(L*,a*,b*)=\sqrt{{\left(100-L\right)}^{2}+{\left(a*\right)}^{2}+{\left(b*\right)}^{2}}\;$$

The discoloration properties of permutite samples were evaluated by the total color difference, as given by Equation (2) [19]:

(2) $$\Delta E=\sqrt{{\left(L_{0}^{*}-L_{1}^{*}\right)}^{2}+{\left(a_{0}^{*}-a_{1}^{*}\right)}^{2}+{\left(b_{0}^{*}-b_{1}^{*}\right)}^{2}}\;$$

The nitrogen adsorption-desorption isotherms were measured by the volumetric method on an automatic adsorption instrument (ASAP 2010, Micromeritics, Norcross, GA, USA). The pore structure of permutite samples was analyzed by means of nitrogen sorption at 77.36 K. Specific surface area was calculated by the Brunauer-Emmett-Teller (BET) method based on the data in the p/p_0_ range between 0.05 and 0.25. A Barrett-Joyner-Halenda (BJH) analysis was carried out to calculate the mesopore volumes.

### 2.5. FT-IR Spectra and DSC Curve of Permutite Samples

Fourier transform infrared spectroscopy (FT-IR, Nicolet 6700, White Bear Lake, MN, USA) was used to determine the presence of material and chemical interactions. For CEO and permutite powder samples, Potassium bromide (KBr) disks were adopted for FT-IR measurement. The FT-IR spectra analysis was performed in the mean infrared region with a wave number range of 4000–500 cm^−1^ and a spectral resolution of 4 cm^−1^. The signals were processed using the OMNIC spectroscopic software (OMNIC8.0, Nicolet 6700, White Bear Lake, MN, USA). The thermal analysis of CEO and permutite samples was carried out by DSC (Q20, TA Instruments, New Castle, DE, USA), and were performed from 20 °C to 300 °C, at a heating rate of 10 °C/min, under nitrogen atmosphere at a flow rate of 20 mL/min.

### 2.6. The Loading Capacity and Release Behavior

The loading capacity (LC) of CEO was determined as the quantity of CEO encapsulated in Ag^+^/Zn^2+^-permutite. The calculation is presented as Equation (3):

(3) $$LC\left(\%\right)=\frac{Amount{\;\mathrm{of}\;\mathrm{oil}\;\mathrm{content}\;\mathrm{in}\;\mathrm{the}\;\mathrm{Ag}}^{+}/{\mathrm{Zn}}^{2+}-\mathrm{permutite}}{Weight{\;\mathrm{of}\;\mathrm{the}\;\mathrm{Ag}}^{+}/{\mathrm{Zn}}^{2+}-\mathrm{permutite}/\mathrm{CCEO}}\times 100\%\;$$

The calibration curve of the CEO in the ethanol solution was first carried out by UV-Vis spectrophotometer (N4, Lengguang, Shanghai, China) at 305 nm (the maximum wavelength). The quantity of CEO encapsulated within Ag^+^/Zn^2+^-permutite was determined using solvent extraction. About 267.4 mg of Ag^+^/Zn^2+^-permutite/CEO was dissolved in 25 mL ethanol using a 50 mL beaker for 10 min to ensure complete dispersal of the Ag^+^/Zn^2+^-permutite/CEO. The supernatant liquid was collected in a flask and the CEO content was determined by measuring the absorbance at 305 nm in triplicate.

To study the release behavior of CEO encapsulated in Ag^+^/Zn^2+^-permutite in air, 2.0 g Ag^+^/Zn^2+^-permutite/CEO was stored at different temperatures (4 °C, 15 °C and 25 °C) in air for 18 days. The amount of CEO released at each specified storage period, was determined in triplicate.

### 2.7. Antimicrobial Assays

Strains of *Aspergillus niger* (ATCC6725) and *Penicillium* (ATCC1109) were provided by the Department of Microbiology of Zhejiang University of Technology. The cultivation medium, potato dextrose agar (PDA), was prepared in line with the manufacturer’s instructions, following which 7.8 g was measured into Erlenmeyer flasks (500 mL) and 200 mL sterile water was added to the different flasks and the contents were slightly heated in a microwave oven for the proper dissolution of the mixture. They were sterilized at 121 °C for 15 min. As diluents, distilled water with 0.1% Tween 80 and physiological solution (NaCl 0.9%) were used.

The ethyl cellulose solutions (10 wt%) were prepared by dissolving 2.0 g ethyl cellulose powder into ethanol under constant stirring using a magnetic stirrer (topolino, IKA, Berlin, German) for 30 min. Then, 100 mg of permutite sample was dissolved in ethyl cellulose solution under constant stirring at room temperature for 1 h. For comparison, a solution of pure ethyl cellulose was also prepared. The pads were prepared using a syringe to draw 2 mL dispersion to apply evenly to absorbent paper. Potatoes (200 g) were peeled and cut into small pieces, and 1000 mL of water was added, letting it boil for 25–30 min, and then gauze was used to filter it. Finally, 20 g glucose and 15 g agar were added, and then it was steamed in a 0.07 MPa autoclave. The antimicrobial activity of the ion-exchanged permutite pads was investigated against *Aspergillus niger* and *Penicillium citrinum* according to G21-96 (2002) Standard Practice for Determining Resistance of Synthetic Polymeric Materials to Fungi [20,21]. The pads were cut into 50 mm × 50 mm squares, the growth of fungi on the antimicrobial pad was observed.

### 2.8. Extension of the Shelf Life of Chinese Bayberry Fruit and Migration Security Tests

Four treatment groups of Chinese bayberries (*Myrica rubra*) were treated with different pads (a: the ethyl cellulose pads, as control; b: the ethyl cellulose pads modified by permutite, c: the ethyl cellulose pads modified by Ag^+^/Zn^2+^-permutite, d: the ethyl cellulose pads modified by Ag^+^/Zn^2+^-permutite/CEO). All groups were packed in PE plastic boxes and stored at 15 ± 0.5 °C; the changes of Chinese bayberry after storage at 0, 24, 48, 72, 96 h were observed. The fruit rots were expressed as the percentage of the rotten area. The decay rate was calculated using the following Equation (4):

(4) $$Decay\;rate\left(\%\right)=\left(\frac{Rotten\;\mathrm{area}\;\mathrm{of}\;\mathrm{fruit}}{\mathrm{Total}\;\mathrm{area}}\right)\times 100\%\;$$

The total soluble solids (TSS) of the fruit was determined with a hand-held refractometer in duplicate (Chengdu Optical Instrument Factory, Chengdu, China). Chinese bayberry fruit decay was visually evaluated immediately at the end of storage (4 days) in air to simulate retail market conditions [22].

The total specific migration limit (SML) of metal ions was measured by exposure of the bimetallic Ag^+^/Zn^2+^-permutite antimicrobial pads to the simulants (distilled water). The pads were cut into 38.47 cm^2^ pieces and immersed in 250 mL simulant in a conical flask for 10 days at 40 °C. Then the supernatant was collected and analyzed for Ag^+^, Zn^2+^ and Al^3+^ concentration using ICP-MS (ELAN DRC-e, PerkinElmer, Waltham, MA, USA). Briefly, in a 50 mL digestion vessel, 200 mg of freeze-dried sample was mixed with 3 mL of HNO_3_ and 1.5 mL of H_2_O_2_ and digested in a Mars5 microwave oven (CEM, Matthews, NC, USA). The digested sample solution was transferred to 50 mL polypropylene tube; the vessel was sparingly rinsed with a small amount of deionized water (18.2 MΩ cm) several times. The aliquots were pooled and diluted to 30 mL using deionized water. The concentrations of arsenic, cadmium, mercury and lead were determined by ICP-MS. The indium nuclide (115In) was used as an internal standard.

### 2.9. Statistical Analysis

The statistical analyses were carried out using Origin pro 8.5 (Originlab, Northampton, Massachusetts, MA, USA); statistical differences were determined using a one-way analysis of variance (ANOVA) with values of *p* < 0.05 considered to be statistically significant.

## 3. Results

### 3.1. Permutite Sample Characterization

**Figure 2:**
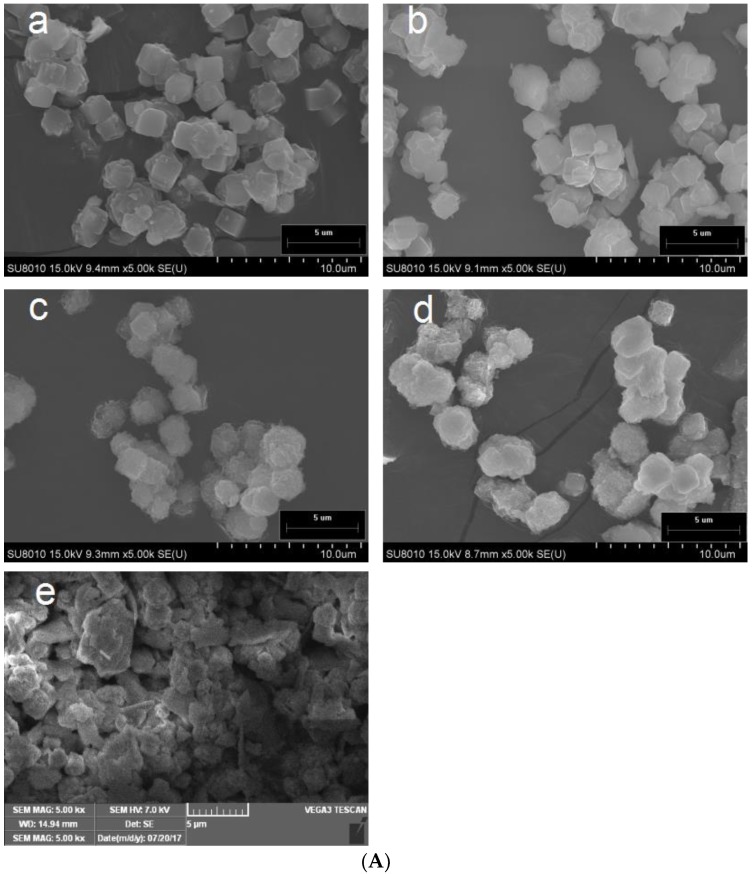

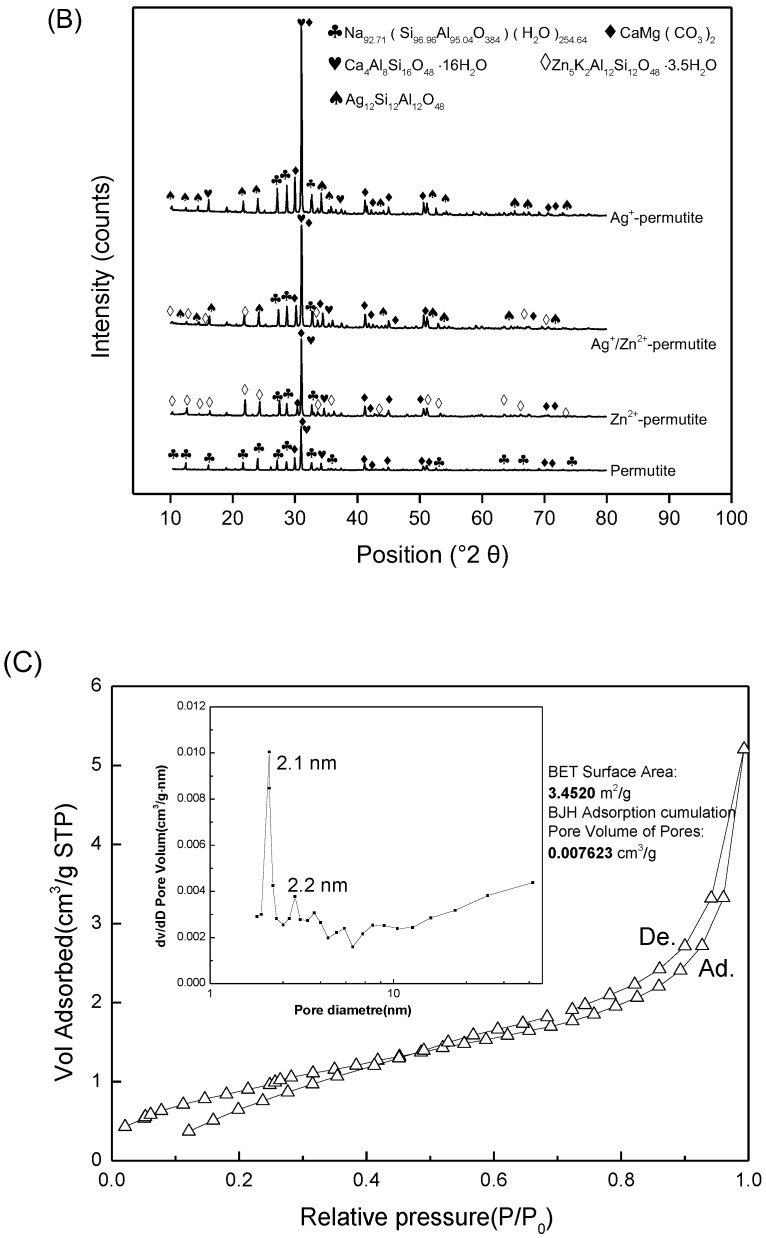

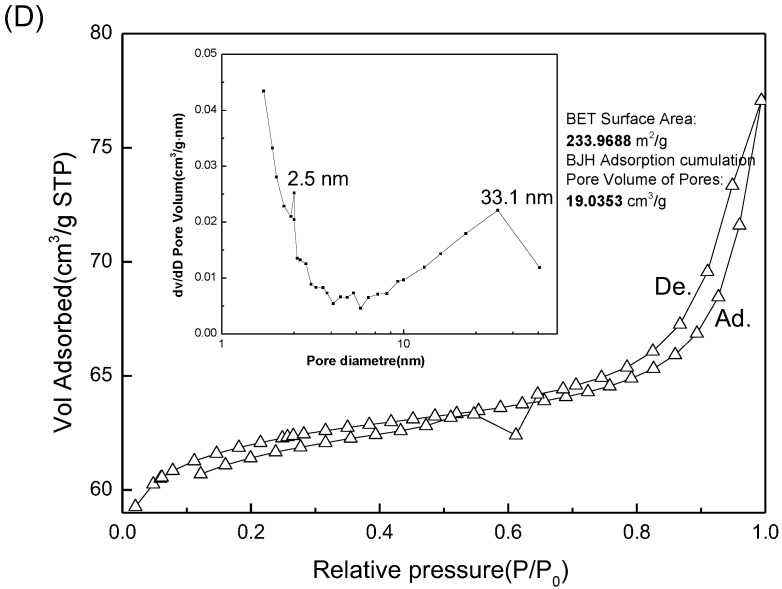
SEM images (**A**) and XRD spectra (**B**) of permutite with different treatments; (a) Untreated, (b) 0.1 mol Ag^+^, (c) 0.1 mol Zn^2+^, (d) 0.1 mol Ag^+^ and 0.1 mol Zn^2+^; Nitrogen sorption isotherms of permutite samples; (**C**) permutite, (**D**) Ag^+^/Zn^2+^-permutite.

The morphology of the permutite after ion exchange was studied by SEM and is shown in Figure 2A. Both the morphologies and particle sizes of all permutite samples were similar, consisting of cubic particles from 4.3 to 6.2 µm. Treated with 0.1 mol L^−1^ concentrations of AgNO_3_ solution, the crystal morphology of the permutite was not significantly changed. However, the same concentration of Zn^2+^ solution caused defects to the permutite crystal. These results demonstrated that the ion exchange method had modified the morphology of the permutate [23]. Table 1 shows the amounts of Ag^+^, Zn^2+^, K^+^, Mg^2+^, Na^+^ and Ca^2+^. The results show that K^+^ and Na^+^ in the permutite were easily displaced by other metal ions. This is due to the ionic radii and charge differences among four ions, with the sizes of the ion radii being, from largest to smallest: K^+^ (+1, 0.133 nm) > Ag^+^ (+1, 0.115 nm) > Ca^2+^ (+2, 0.099 nm) > Na^+^ (+1, 0.097 nm) > Zn^2+^ (+2, 0.074 nm) > Mg^2+^ (+2, 0.072 nm). In general, the smaller the charge and the greater the ion radius, the easier it is for ions to be replaced by other ions from the permutite.

X-ray diffraction (XRD) was used to evaluate possible modifications in the permutite structure during the ion exchange process (Figure 2B). The permutite structure was preserved in all the monometallic and bimetallic materials prepared, as confirmed by XRD analysis. As expected, all diffraction peaks in the powder XRD patterns of the materials were very similar and exhibited the typical and comparable pattern of the parent LTA zeolite structure (PDF: 089-5423) [24]. According to the XRD route, permutite has a phase formula of Na_92.71_(Si_96.96_Al_95.04_O_384_)∙H_2_O_254.64_, CaMg(CO_3_)_2_ and Ca_4_Al_8_Si_16_O_48_∙16H_2_O [7]. The properties of permutite may be similar to those of natural zeolite.

Silver ions are very sensitive to UV irradiation [25]. The Ag^+^-permutite and Zn^2+^-permutite showed a similar *W* (L*, a*, b*) before UV irradiation. After 254 nm UV irradiation, it was peculiar that Ag^+^-permutite pads showed a higher whiteness [26]. The total color difference of Ag^+^/Zn^2+^-permutite was lower than that of Ag^+^-permutite pads (*p* < 0.05). These results suggested that with the addition of zinc ion, the anti-discoloration property of the pad had been remarkably improved (Table 2). This is due to two reasons: the first is that silver ions are very sensitive to UV irradiation, while zinc ions are not [27]. Another reason is that in the ion exchange process, zinc ions can reduce the number of silver ions in the permutate [28,29].

Nitrogen sorption is used to determine the specific surface area and the distribution of aperture [30]. The nitrogen adsorption/desorption isotherms of the permutite and Ag^+^/Zn^2+^-permutite are illustrated in Figure 2C,D. The sorption of the Ag^+^/Zn^2+^-permutite is higher than that of the permutite, proving that the porosity of permutite treated with Ag^+^/Zn^2+^ solution was much higher than that of untreated permutite. The BET specific surface area and pore volume of the samples were calculated from their adsorption data. The BET specific surface area of permutite was 34,520 m^2^/g, while the surface area of Ag^+^/Zn^2+^-permutite was 2,339,688 cm^2^/g. The BJH pore volume of permutite was 0.007623 cm^3^/g, while the surface area of Ag^+^/Zn^2+^-permutite was 19.0353 cm^3^/g. The BJH analysis implies that there was a large quantity of mesopores in the permutite and Ag^+^/Zn^2+^-permutite. After ion-exchange, the pores in the permutite shift from 2.1 and 2.2 nm radius to 2.5 and 33.1 nm, respectively. Meanwhile, Ag^+^/Zn^2+^-permutite had a broad distribution of pore sizes. The isotherm of the Ag^+^/Zn^2+^-permutite exhibited a larger pore volume and surface area compared to permutate [31]. This can be attributed to the silver zinc solution effect, which reduced the soluble solids in permutite. Consequently, organic molecules can diffuse to the Ag^+^/Zn^2+^-permutite crystals with a smaller resistance [32].

### 3.2. Characterization of Ag^+^/Zn^2+^-Permutite/CEO

**Figure 3:**
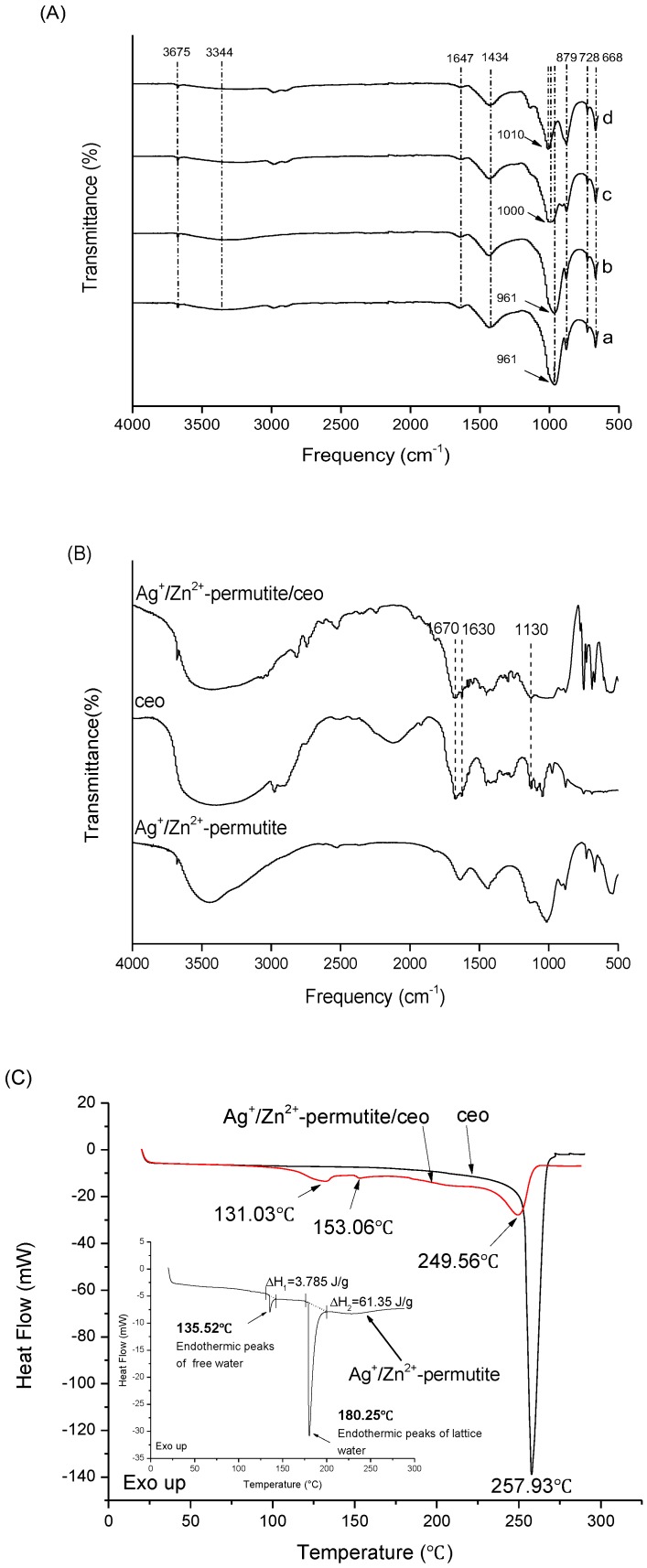
FTIR spectra (**A**) of ion-exchanged permutite: (a) permutite, (b) Ag^+^-permutite, (c) Ag^+^/Zn^2+^-permutite, (d) Zn^2+^-permutite; FTIR spectra (**B**) of Ag^+^/Zn^2+^-permutite/CEO; DSC curve (**C**) of permutite samples.

The ion-exchanged permutite samples were characterized by FTIR, and the results are shown in Figure 3A. The peaks at 3675 cm^−1^ and 3344 cm^−1^ are attributed to the telescopic vibration of hydroxyl (lattice water in β cage of permutite) and the vibration of hydroxyl (free water in α cage of permutite) (Figure 3C). This indicates that the permutite contains two forms of water. It further demonstrates that the permutite structure has pores that can accommodate antimicrobial substances. The peaks at 1647 cm^−1^, 1434 cm^−1^, 879 cm^−1^, 728 cm^−1^, 668 cm^−1^ are characteristic peaks of permutite, which were attributed to the silicon (aluminum) oxygen tetrahedral skeleton vibration, asymmetric stretching vibration of CO_3_^2−^, stretching vibration of Al–O–Al, symmetrical vibration of Si–O–Si and symmetrical stretching vibration of Si–O–Si (Al), respectively. Compared to the other permutite samples, the peaks at 2988 cm^−1^ and 2900 cm^−1^ disappeared in the Ag^+^-permutite sample. The asymmetric stretching vibration peak of Si–O–Si and Si–O–(Al) at 965 cm^−1^ received the effects of Ag^+^ and Zn^2+^, and their corresponding peaks were red shifted (1006 cm^−1^) [33]. FT-IR spectroscopy helps to reveal the presence of functional groups and possible interactions between CEO and Ag^+^/Zn^2+^-permutite. Figure 3B shows the infrared spectra of the fibrous film and the infrared spectra of the CEO. The C=O stretching vibration around 1672 cm^−1^ and the benzene ring skeleton vibration around 1625 cm^−1^ are the characteristic peaks of CEO. These results indicate that there was no molecular interaction between CEO and Ag^+^/Zn^2+^-permutite.

The DSC thermogram (Figure 3C) of pure CEO shows that CEO started to evaporate and degraded at 257.93 °C, which could be interpreted as an exothermic response. Ag^+^/Zn^2+^-permutite lost their water in two steps, namely at 135.52 °C and 180.25 °C, which corresponds to free water and lattice water. However, the thermogram of Ag^+^/Zn^2+^-permutite/CEO showed endothermic peaks (131.03 °C, 153.06 °C) corresponding to the evaporation of CEO from Ag^+^/Zn^2+^-permutite. In comparison, because the CEO could evaporate with water vapor, CEO in Ag^+^/Zn^2+^-permutite shows a lower boiling point than pure essential oils.

### 3.3. Release Behavior of Ag^+^/Zn^2+^-Permutite/CEO

**Figure 4:**
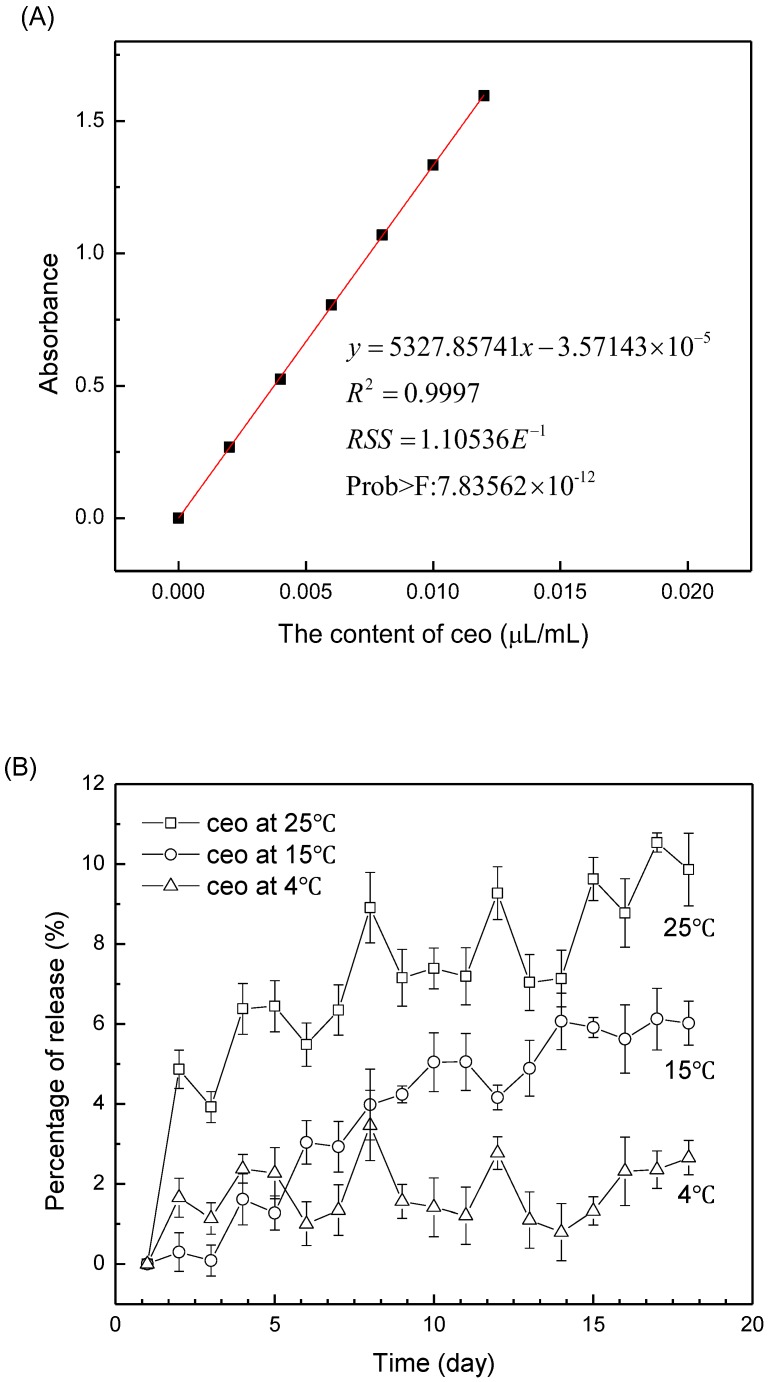

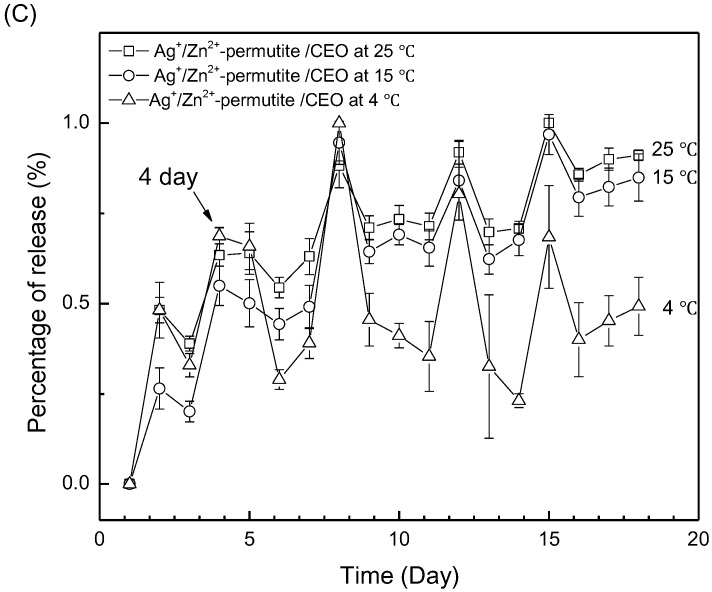
Standard curve (**A**) of cinnamon essential oil ethanol solution; the release profiles of CEO (**B**) and Ag^+^/Zn^2+^-permutite/CEO (**C**) storage at different temperatures (4 °C, 15 °C, 25 °C).

The calibration curve of the CEO ethanol solution is shown in Figure 4A. The loading capacity (LC) of Ag^+^/Zn^2+^-permutite/CEO is 313.07 µL/g. The release rate of essential oil increased with the increase of temperature (Figure 4B). Permutite has a stable crystal structure, the interior of which has rich pores and cages; the kinetics of CEO release from the Ag^+^/Zn^2+^-permutite at different temperature (4 °C, 15 °C and 25 °C) is shown in Figure 4C. The results reveal that the CEO was released continually at 4 °C, 15 °C and 25 °C. In the air, the amount of the released CEO from the Ag^+^/Zn^2+^-permutite was less than 2%. In comparison, we observed that the CEO release was much faster than for those encapsulated within the Ag^+^/Zn^2+^-permutite for the same period. These results suggest that the Ag^+^/Zn^2+^-permutite/CEO has a sustained release effect.

### 3.4. Antimicrobial Activity of Pads

**Figure 5:**
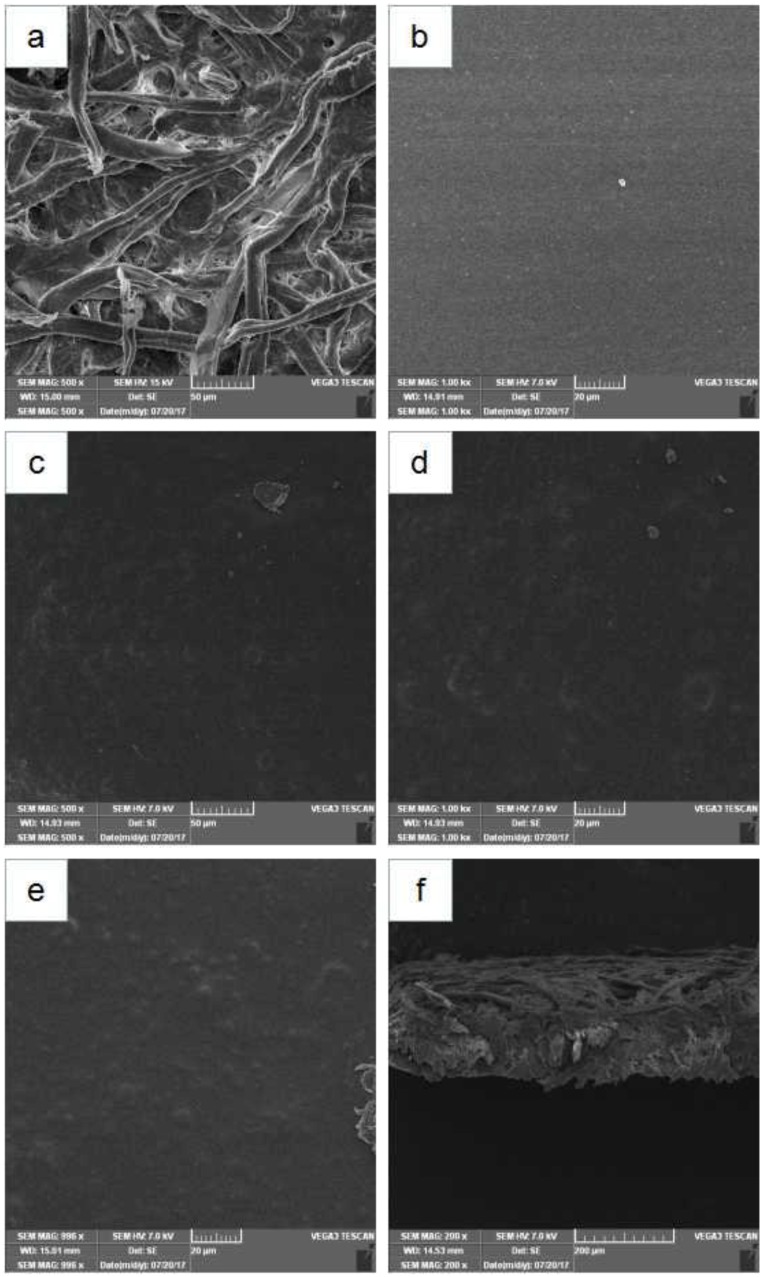
SEM images of antimicrobial pads, (**a**) Absorbent pads (surface), (**b**) The ethyl cellulose pads (surface), (**c**) The ethyl cellulose pads modified by permutite (surface), (**d**) The ethyl cellulose pads modified by Ag^+^/Zn^2+^-permutite (surface), (**e**) The ethyl cellulose pads modified by Ag^+^/Zn^2+^-permutite (surface), (**f**) The ethyl cellulose pads modified by Ag^+^/Zn^2+^-permutite (cross-sectional).

The microstructure of the ethyl cellulose pads modified by permutite samples was studied under SEM. The absorbent paper has a porous microstructure and is a good carrier for the coating, as a rough surface was observed (Figure 5a) in contras to the uniform smooth surface of pure EC pads (Figure 5b). The micro-graphics of the pads modified by permutite samples (Figure 5c–e) showed a rather uniform permutite distribution with certain agglomerations throughout the composites [34]. These effects might be caused by interface incompatibility and a defective nanocomposite structure. Llorens et al. 2012 reported similar defects in the distribution of silver zeolites in polylactide, together with voids growing around the permutate [35]. These results indicate that the composite antimicrobial particles were still acceptably well dispersed in the ethyl cellulose layer.

**Figure 6:**
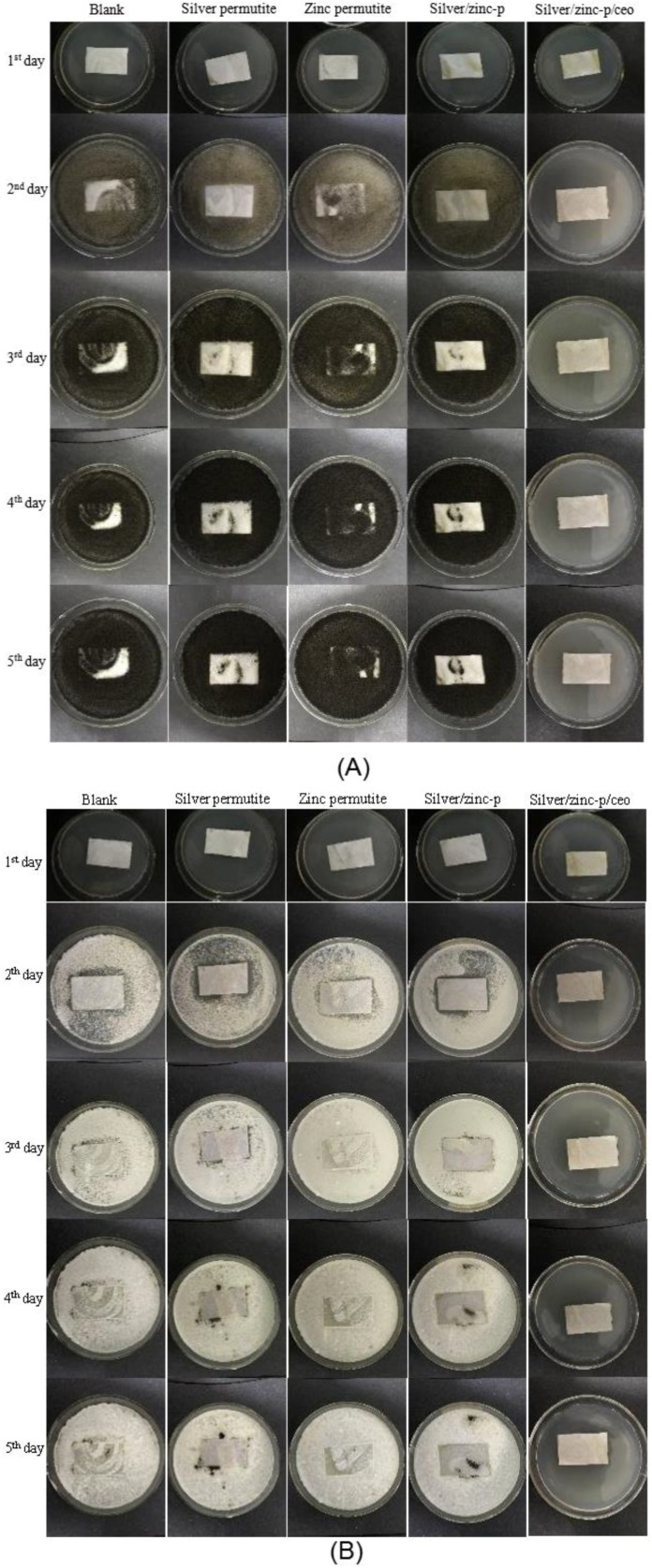
Antimicrobial activity assays against (**A**) *Aspergillus niger* and (**B**) *Penicillium.*

The antimicrobial effects of Ag^+^/Zn^2+^-permutite/CEO were investigated according to the Standard Practice for Determining Resistance of Synthetic Polymeric Materials to Fungi against *Aspergillus niger* and *Penicillium* sp. (Figure 6 and Table 3). According to the observation, pads modified by different permutite samples exhibited varying antimicrobial activity against *Aspergillus niger* and *Penicillium* sp. Among them, pads modified by permutite showed no antimicrobial effect against fungi. Pads modified by Ag^+^/Zn^2+^-permutite showed a poor antimicrobial effect against fungi due to the poor diffusion properties of metal ions. Similar to with other research [36], pads modified by Ag^+^/Zn^2+^-permutite/CEO showed good antimicrobial effect. The antimicrobial activity of the materials was due to the synergistic effect between the CEO and Ag^+^/Zn^2+^ (*p* < 0.05).

### 3.5. Application of the Antimicrobial Pads in Chinese Bayberry Fruit

**Figure 7:**
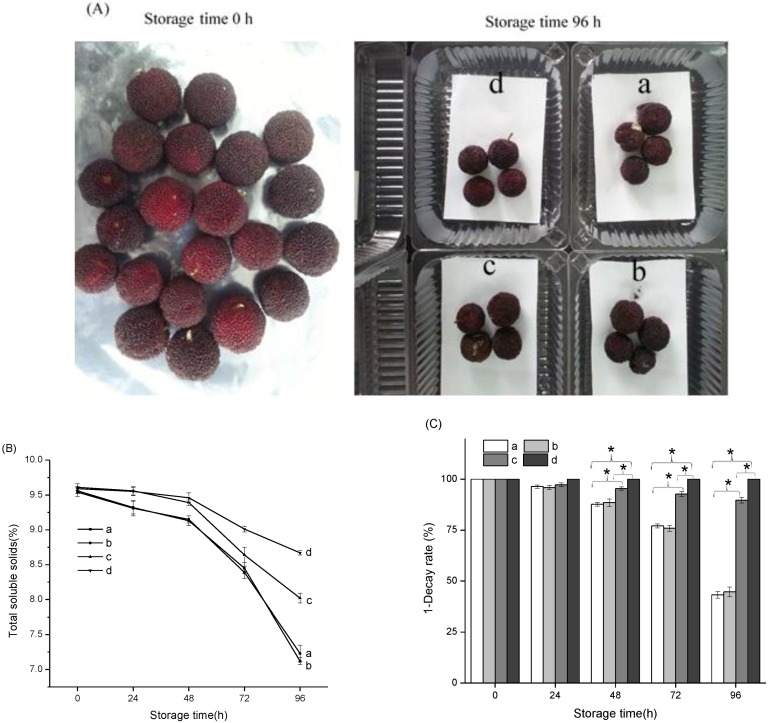
Chinese bayberry fruit at different storage times with different treatments (**A**); Total soluble solids (**B**) and Decay rate (**C**) of Chinese bayberry fruit packaged with different pads; a: The ethyl cellulose pads, as control, b: The ethyl cellulose pads modified by permutite, c: The ethyl cellulose pads modified by Ag^+^/Zn^2+^-permutite, d: The ethyl cellulose pads modified by A^g+^/Zn^2+^-permutite/CEO; * means significantly different at *p* < 0.05 level.

Chinese bayberry is an evergreen tree originating in southeastern China, the fruit of which can be easily damaged by microorganisms, and which has a storage or shelf life of only about 3–4 days at ambient temperatures [37,38]. Normally, the total soluble solids of ripe Chinese bayberry decrease gradually with the extent of storage time [39,40]. As shown in Figure 7B, the total soluble solids (TSS) value of Chinese bayberry storage at 0 h was 9.6%. After storage for 96 h, the TSS value of the control group had decreased to 7.12%, while that of fruits treated with the Ag^+^/Zn^2+^-permutite/CEO pads had decreased to 8.43%. The decay rate clearly reflects the prolonged shelf life effect of the pads on Chinese bayberry fruit. The results presented in Figure 7C show that treatment with the Ag^+^/Zn^2+^-permutite/CEO pads resulted in a significantly lower decay incidence compared to the control group from 48 h, which is consistent with other research [41,42]. Treated with the Ag^+^/Zn^2+^-permutite/CEO pads, the loss rate of Chinese bayberry was lower than 5%.

Migration testing is an important procedure in food packaging, because there is the possibility of migration of components (e.g., monomers or additives) from the packaging to the conditioned product, which is a relevant matter for human health due to potential health-related risks [43]. In the test, the Ag^+^/Zn^2+^-permutite/CEO was added in an amount of 10 mg per 0.5207 g of packaging substrate. The total specific migration limits (SML/T) of the pads are showed in Table 4. The migration of silver ions and zinc ions was 0.04330 mg/L and 0.9420 mg/L in a typical simulant, which is below the legal limit [44]. Moreover, no aluminum ions were released from the permutite, since permutite is an artificial nano-porous alumina silicate composed of silicon, aluminum and oxygen in a framework with cations and water within the pores. The basic structure of zeolite is that of a silicon oxy tetrahedron. Silicon atoms can be replaced by aluminum atoms to form an aluminum oxygen tetrahedron. Therefore, aluminum atoms are more closely combined in zeolite and, for that reason, in the testing of migration, aluminum atoms cannot be released. These results suggest that antimicrobial particles that were completely encapsulated in the host polymer matrix did not have the potential to migrate into the food [45].

## 4. Conclusions

This study provides a new antibacterial agent based on nano-porous permutite particles with a sustained release effect. The Ag^+^/Zn^2+^-permutite was prepared by ion-exchange methods, and the samples were characterized by SEM, XRD, Colorimeter and Nitrogen sorption technique. With the addition of zinc ions, the anti-discoloration property of permutite samples improved remarkably. The isotherm of the Ag^+^/Zn^2+^-permutite exhibited larger pore volume and surface area compared to permutite. On this basis, the CEO was encapsulated in Ag^+^/Zn^2+^-permutite. The FTIR and DSC showed that CEO occupies the pores in the Ag^+^/Zn^2+^-permutite, and there were no molecular interactions between CEO and Ag^+^/Zn^2+^-permutite. The loading capacity (LC) of Ag^+^/Zn^2+^-permutite/CEO was 313.07 µL/g. The Ag^+^/Zn^2+^-permutite/CEO has a sustained release effect and showed good antimicrobial effects. Ethyl cellulose pads modified by composite antimicrobial particles were applied in the preservation of Chinese bayberry. Treatment with the antimicrobial pads resulted in significantly lower decay incidence compared to the control group. In addition, migrated silver, zinc and aluminum in the simulant (distilled water) were quantified using inductively coupled plasma mass spectrometer (ICP-MS). These results confirmed that the ethyl cellulose pads modified by the Ag^+^/Zn^2+^-permutite/CEO provided an effective and safe method to control decay of fresh Chinese bayberry fruit.

## Figures and Tables

**Table 1:** The content of metal ions in ion-exchanged permutite.

Samples	Content of Metal Ion (wt%)					
	Na^+^	Mg^2+^	K^+^	Ca^2+^	Ag^+^	Zn^2+^
a	11.73 ± 0.5	1.35 ± 0.09	0.40 ± 0.03	0.77 ± 0.05	ND	ND
b	6.16 ± 0.3	0.52 ± 0.06	ND	0.49 ± 0.02	18.47 ± 1.32	ND
c	ND	0.87 ± 0.04	ND	0.69 ± 0.04	ND	9.55 ± 0.74
d	1.10 ± 0.3	0.65 ± 0.03	ND	0.47 ± 0.06	9.68 ± 0.94	7.80 ± 0.61

Permutite with different treatments; (a) Untreated, (b) 0.1 mol Ag^+^, (c) 0.1 mol Zn^2+^, (d) 0.1 mol Ag^+^ and 0.1 mol Zn^2+^; ND, not detected; Samples were tested in triplicate and the mean values were calculated.

**Table 2:** Anti-discoloration test of ion-exchanged permutite.

Specimens	L*		a*		b*		*W* (*L*, *a*, *b*)		*∆E*
	Original	UV	Original	UV	Original	UV	Original	UV	
a	91.26 ± 2.42	88.20 ± 3.62	0.00	−0.09 ± 0.02	−0.38 ± 0.06	−0.48 ± 0.09	91.3 ± 3.98	88.2 ± 2.13	3.1 ± 0.41
b	72.04 ± 1.42	82.08 ± 2.41	1.00 ± 0.12	−0.42 ± 0.04	5.08 ± 0.24	4.45 ± 0.29	71.6 ± 2.12	81.5 ± 2.94	10.2 ± 0.67
c	76.65 ± 2.38	74.95 ± 1.46	−0.17 ± 0.06	−0.40 ± 0.04	0.23 ± 0.06	0.73 ± 0.14	76.6 ± 2.65	74.9 ± 2.45	1.8 ± 0.27
d	71.99 ± 2.23	77.72 ± 1.77	0.45 ± 0.09	−0.10 ± 0.01	2.53 ± 0.14	2.22 ± 0.19	71.9 ± 2.71	77.6 ± 2.38	5.8 ± 0.38

Permutite treated with: (a) Untreated, (b) 0.1 mol Ag^+^, (c) 0.1 mol Zn^2+^, (d) 0.1 mol Ag^+^ and 0.1 mol Zn^2^^+^, UV means 254 nm UV irradiation 4 h, cycle 5 times, different superscripts within the same column indicate significant difference between formulations (*p* < 0.05. Samples were tested in triplicate and the mean values were calculated).

**Table 3:** Evaluation table of mold growth.

Time (d)	Control		Silver Permutite		Zinc Permutite		Silver/Zinc Permutite		Silver/Zinc p-CEO	
	A	P	A	P	A	P	A	P	A	P
1	0	0	0	0	0	0	0	0	0	0
2	2	2	1	2	1	2	1	2	0	0
3	3	3	2	2	2	3	1	2	0	0
4	4	4	2	3	3	3	2	3	0	0
5	4	4	3	4	3	4	2	3	0	0

A stands for *Aspergillus niger*; P stands for *Penicillium*; d stands for days. Growth ratings: 0, no growth; 1, traces of growth (less than 10%); 2, light growth (10% to 30%); 3, medium growth (30% to 60%); 4, heavy growth (60% to complete coverage).

**Table 4:** The total specific migration limits (SML/T) of Ag^+^/Zn^2+^-permutite/ceo pads.

Simulants	Ions Migrate from Ag^+^/Zn^2+^-Permutite/CEO Antibacterial Pads (mg/L)		
	Ag^+^	Zn^2+^	Al^3+^
Water	0.04330 ± 0.002	0.9420 ± 0.005	ND
Limit value	0.5	25	0.2

Pads immersed in 250 mL simulants in conical flask for 10 days at 40 °C. ND means not detected.
